# UL45 of Human Cytomegalovirus Promotes Macrophage Survival and Modulates RIPK3-Associated Cell Death

**DOI:** 10.3390/v18091033

**Published:** 2026-09-17

**Authors:** Christian S. Yu, Komal Beeton, Lisa P. Daley-Bauer, Stephen J. Stray

**Affiliations:** 1 Center for Immunology and Microbial Research, Jackson, MS 39216, USA; csyu@umc.edu (C.S.Y.); kkomal@bu.edu (K.B.); 2 Department of Cell and Molecular Biology, University of Mississippi Medical Center, Jackson, MS 39216, USA; 3 Pathology Advanced Translational Research Unit, Department of Pathology and Laboratory Medicine, Emory University, Atlanta, GA 30322, USA

**Keywords:** necroptosis, UL45 protein, human cytomegalovirus

## Abstract

Programmed necrotic cell death (necroptosis) is a key antiviral defense pathway, yet its regulation during human cytomegalovirus (HCMV) infection remains incompletely defined in physiologically relevant cell types. The M45 protein of murine cytomegalovirus, a homologue of UL45, inhibits necroptosis and contributes to MCMV dissemination, providing a rationale for examining UL45 in HCMV. Previous studies of UL45 have largely relied only on fibroblasts or immortalized cell lines, which lack the necroptotic machinery necessary to reveal viral anti-necroptotic functions. To investigate the role of the HCMV UL45 protein, we generated a UL45 stop-frameshift mutant (UL45st) in the TB40E strain and compared its phenotype to that of the wild-type virus in primary human fibroblasts and monocyte-derived macrophages. In parallel, transfection-based assays in RIPK3-competent HT29 cells were used to assess the intrinsic cell-death-inhibitory activity of UL45. UL45st-infected primary macrophages exhibited significantly reduced numbers of viable cells and showed morphological features suggesting necroptotic death compared to wild-type-infected cells, whereas infected fibroblasts showed only modest defects. Expression of UL45 alone in HT29 cells partially protected cells from both apoptotic and RIPK3-dependent necroptotic stimuli, indicating a direct anti-death function of the protein. These findings demonstrate that HCMV UL45 promotes cellular survival in primary human macrophages and reveal that this function is likely associated with RIPK3-associated cell death. Collectively, this work identifies a never-before-shown function of HCMV UL45 as a key determinant of viral survival in myeloid compartment cells and underscores the importance of using physiologically relevant models to uncover cell-type-specific viral immune evasion mechanisms.

## 1. Introduction

Viruses must contend with multiple programmed cell death (PCD) pathways triggered upon infection of host cells, and many viruses have evolved strategies to subvert these innate immune pathways to ensure their survival [1,2]. Apoptosis is a regulated form of PCD characterized by membrane blebbing, with maintenance of cell membrane integrity and little to no inflammation [3]. Apoptosis can be triggered by an extrinsic TNFR1 death receptor signal, an intrinsic p53 DNA damage signal, an intrinsic cGas/STING viral DNA sensing signal, or an intrinsic lack-of-survival Bcl-2 signal, and is mediated by the proteolytic activation of cysteine-aspartic proteases known as caspases [4]. Necroptosis is a regulated form of necrosis characterized by loss of membrane integrity, leakage of cellular contents, and consequent recruitment of immune effector cells [5,6]. Necroptosis plays important roles in inflammation, cancer, and the prevention of viral and bacterial infections [7]. Notably, both apoptosis and necroptosis can be triggered by the extrinsic TNFR1 death receptor signal; apoptosis depends on proteolytic activation of caspases, while inhibition of caspase activity diverts signaling toward receptor-interacting protein kinase (RIPK)-mediated necroptosis [8]. Necroptosis can also be triggered independently through intrinsic toll-like receptor (TLR) 3 signaling or direct sensing of cytoplasmic viral DNA by DNA-binding proteins during viral infections [5,8]. The key molecular executors of necroptosis are RIPK1, RIPK3, and mixed-lineage kinase domain-like protein (MLKL) [9,10]. Despite significant advances, the mechanisms by which necroptosis shapes the outcome of HCMV infection remain incompletely defined, particularly in the cell types most relevant to infection in vivo.

### 1.1. HCMV Effects on Host Cell Death Machinery

As with many other viruses, antagonizing PCD pathways is essential for both HCMV replication and dissemination [11]. HCMV has been shown to inhibit caspase-dependent, caspase-independent, and autophagic PCD in various cell types [11,12,13,14,15]. HCMV protein UL36 has been identified as the viral inhibitor of caspase-8 activation (vICA), which inhibits apoptosis in infected cells, has been implicated as an inhibitor of caspase-independent cell death in a monocyte cell line, and has been shown to bind and target MLKL for degradation in fibroblasts [13,16]. HCMV lacking the immediate-early protein IE1, necessary for production of late viral gene products, was unable to prevent caspase-independent cell death in RIPK3-expressing fibroblasts, implicating late viral gene products in inhibition of this pathway [14]. HCMV UL45 and UL48 cooperatively inhibit host RIPK1 via protein–protein interactions among the three proteins, with RIPK1 serving as an early mediator of both apoptotic and necroptotic signaling in the TNFR1 death receptor pathway [17].

Due to the lack of an animal model for HCMV, murine CMV (MCMV) is an important model for understanding the role of PCD in CMV infection, especially at the organismal level. MCMV M45, the homolog of HCMV UL45, binds RIPK1, RIPK3, and NEMO and inhibits necroptosis in mice. M45 has been termed the viral inhibitor of RIP activation (vIRA) and is thought to be the major inhibitor of RIPK3-dependent necrotic death in MCMV-infected cells. M45 binds RIPK1 and RIPK3 via a receptor-interacting homotypic interaction motif (RHIM) domain, and binds RIPK1 and NEMO through an interacting protein aggregation motif (IPAM) [18,19,20]. Unlike M45, HCMV UL45 does not contain an N-terminal RHIM domain but does retain a partially conserved C-terminal IPAM motif [11]. UL45 has been shown to be dispensable for growth in endothelial cells and fibroblasts, but UL45 mutants exhibit a defect in growth and cell-to-cell spread in fibroblasts at low multiplicity of infection (MOI). Fibroblasts infected with a UL45 knockout HCMV were also more sensitive to Fas-induced apoptosis than wild-type HCMV-infected cells [21,22]. More recently, UL45 has been shown to colocalize with RIPK1 and UL48 at the viral assembly complex (VAC) during infection and coimmunoprecipitates with RIPK1 in an RHIM-independent manner when both proteins are overexpressed in HEK293T cells [17]. Importantly, however, these studies of HCMV anti-death functions were largely conducted in fibroblasts or immortalized cell lines, a limitation that, as discussed below, has obscured the identification of cell-type-specific anti-necroptotic mechanisms.

### 1.2. Macrophages in HCMV Infection and Immunity

HCMV spreads within the host via infected antigen-presenting cells (APCs) such as macrophages and dendritic cells (DCs), following primary infection or via circulating infected monocytes during secondary infection [11]. Tissue-resident macrophages (TRMs) and DCs are recruited to the site of primary HCMV infection, usually the respiratory tract; become infected; and travel to secondary lymphoid organs, secreting virus along the way. Circulating monocytes in the lymphoid tissues become infected and disseminate the virus to organs throughout the body [23]. HCMV infection of monocytes induces their differentiation into macrophage-like cells that replicate and disperse virus in tissues and organs, and extends their survival well beyond the typical 1–3-day lifespan of circulating monocytes [24]. HCMV-infected monocytes survive for many days, even weeks, because host cell autophagic, apoptotic, and necroptotic signaling is interrupted by viral proteins. Notably, HCMV does not replicate in infected monocytes until the cells have differentiated into macrophages [12,25]. Macrophages infected with HCMV also serve as a reservoir for periodic viral reactivation, playing a role in the maintenance of a large HCMV-specific T-cell repertoire in seropositive individuals [26]. Both pro-inflammatory M1 macrophages and anti-inflammatory M2 macrophages are susceptible to HCMV infection, although it remains unclear whether these subsets play differential roles in viral dissemination or persistence [26,27,28,29].

Inhibition of macrophage necroptosis is a critical determinant of CMV pathogenesis, as demonstrated by the MCMV M45/vIRA protein, which prevents RIPK3-dependent necroptosis and is required for viral dissemination in macrophages, but not for other cell types [19,29,30,31,32]. However, studies of HCMV anti-death mechanisms have largely relied on fibroblasts or immortalized cell lines, which are poorly suited to model necroptosis due to low RIPK3 expression or altered cell death regulation [14]. In contrast, primary human macrophages express high levels of RIPK3 and MLKL and are fully competent for necroptosis, while also playing a very critical role in viral dissemination when extended cellular survival directly translates to increased likelihood of a robust viral infection. These characteristics make macrophages a very relevant system for defining myeloid-specific mechanisms of viral cell death evasion [33]. Based on the structural conservation between MCMV M45 and HCMV UL45, and the reported interaction of UL45 with RIPK1 [17], we hypothesized that UL45 inhibits necroptosis in primary human macrophages. To test this, we generated a UL45 stop-frameshift mutant (UL45st) in the clinical TB40E strain and examined viral replication and cell death phenotypes in monocyte-derived macrophages and necroptosis-competent HT29 cells.

## 2. Materials and Methods

### 2.1. Reagents and Antibodies

Dimethyl sulfoxide (DMSO) and lipopolysaccharide (LPS) were purchased from Sigma (Sigma-Alrich, St Louis, MO, USA). Recombinant human IL-4, IFN-γ, GM-CSF, and M-CSF were purchased from BioLegend (San Diego, CA, USA). IAP antagonist/Smac mimetic BV6 and pan-caspase inhibitor Z-VAD-FMK were purchased from APExBio Technology (Houston, TX, USA). Recombinant human TNF-α was purchased from R&D Systems (Minneapolis, MN, USA). RIP3 kinase inhibitor GSK840 was purchased from MedChemExpress (Monmouth Junction, NJ, USA). The following antibodies were used in cell death assays, immunoblot (IB), and immunofluorescence analysis (IFA): rabbit anti-RIP3 (E1Z1D, Cell Signaling Technology, Danvers, MA, USA), rabbit anti-MLKL (D2I6N, Cell Signaling Technology), rabbit anti-MLKL (phospho-Ser-358) (D6HH3V, Cell Signaling Technology), mouse anti-RIP1 (D94C12, Cell Signaling), mouse anti-FLAG (clone M2, Sigma, see above), anti-Myc (clone 9E10, Santa Cruz Biotechnology, Dallas, TX, USA), mouse anti-human CMV IE1/IE2 (CH160, Virusys, Ijamsville, MD, USA), peroxidase-labeled horse anti-mouse or anti-rabbit IgG (Invitrogen, [Thermo Fisher Scientific], Waltham, MA, USA), Alexa Fluor 488-conjugated goat anti-rabbit IgG (Invitrogen), and Alexa Fluor 568-conjugated goat anti-mouse IgG (Invitrogen).

### 2.2. Cells and Viruses

Primary human foreskin fibroblasts (HFFs) were isolated as described previously [34] and utilized from frozen stocks. HFFs and HT-29s were cultured in Dulbecco’s modified Eagle’s medium (DMEM, Cellgro, Manassas, VA, USA) containing 4.5 g/mL glucose, 10% fetal bovine serum (SAFC, Lenexa, KS, USA), 1 mM sodium pyruvate, 2 mM L-glutamine, and 1% penicillin–streptomycin (Cellgro, Manassas, VA, USA) at 37 °C with 5% CO_2_. HT-29 human colorectal adenocarcinoma cells were obtained from the American Type Culture Collection (ATCC, Manassas, VA, USA). Primary peripheral blood mononuclear cells (PBMCs) were collected from deidentified, discarded blood from healthy donors from the UMMC clinical microbiology laboratory. The study was reviewed by the UMMC Institutional Review Board (IRB) and found to be not human research. Isolated primary peripheral blood mononuclear cells (PMBCs) were cultured in RPMI 1640 (Life Technologies, Grand Island, NY, USA) supplemented with 10% fetal bovine serum and 1% penicillin–streptomycin (Cellgro, Manassas, VA, USA) at 37 °C with 5% CO_2_.

Wild-type TB40E and UL45st mutant HCMV were grown on HFF cells. HFF cells (100% confluent) were infected at a multiplicity of infection (MOI) of 0.01 with either wild-type or mutant virus. Two days after 100% cytopathic effect was observed, cells and supernatant were harvested and frozen at −80 °C, then thawed and sonicated. Cellular debris was removed by low-speed centrifugation. Virus was pelleted from the supernatant by centrifugation at 20,000× *g* for 1 h at 4 °C. Virus stocks were harvested by scraping infected cells and storing in a minimal volume of 3x-autoclaved milk (10% Carnation (Nestlé) instant nonfat dry milk in water) at −80 °C.

Infectious virus titers were determined by plaque assay on HFF cells as previously described [35]. Briefly, serial ten-fold dilutions of virus-containing samples were prepared in serum-free DMEM and inoculated onto confluent monolayers of HFF cells in 6-well plates. After adsorption for 1 h at 37 °C with 5% CO_2_, the inoculum was removed and replaced with complete DMEM containing 0.5% agarose overlay to restrict extracellular viral spread. Plates were incubated at 37 °C for 10–14 days, with a second overlay added at day 7. Monolayers were then fixed with 4% formaldehyde and stained with 0.05% crystal violet to visualize plaques. Viral titers were expressed as plaque-forming units per milliliter (PFU/mL), calculated from wells containing 10–100 countable plaques.

### 2.3. Generation of UL45st Virus

**Figure 1:**
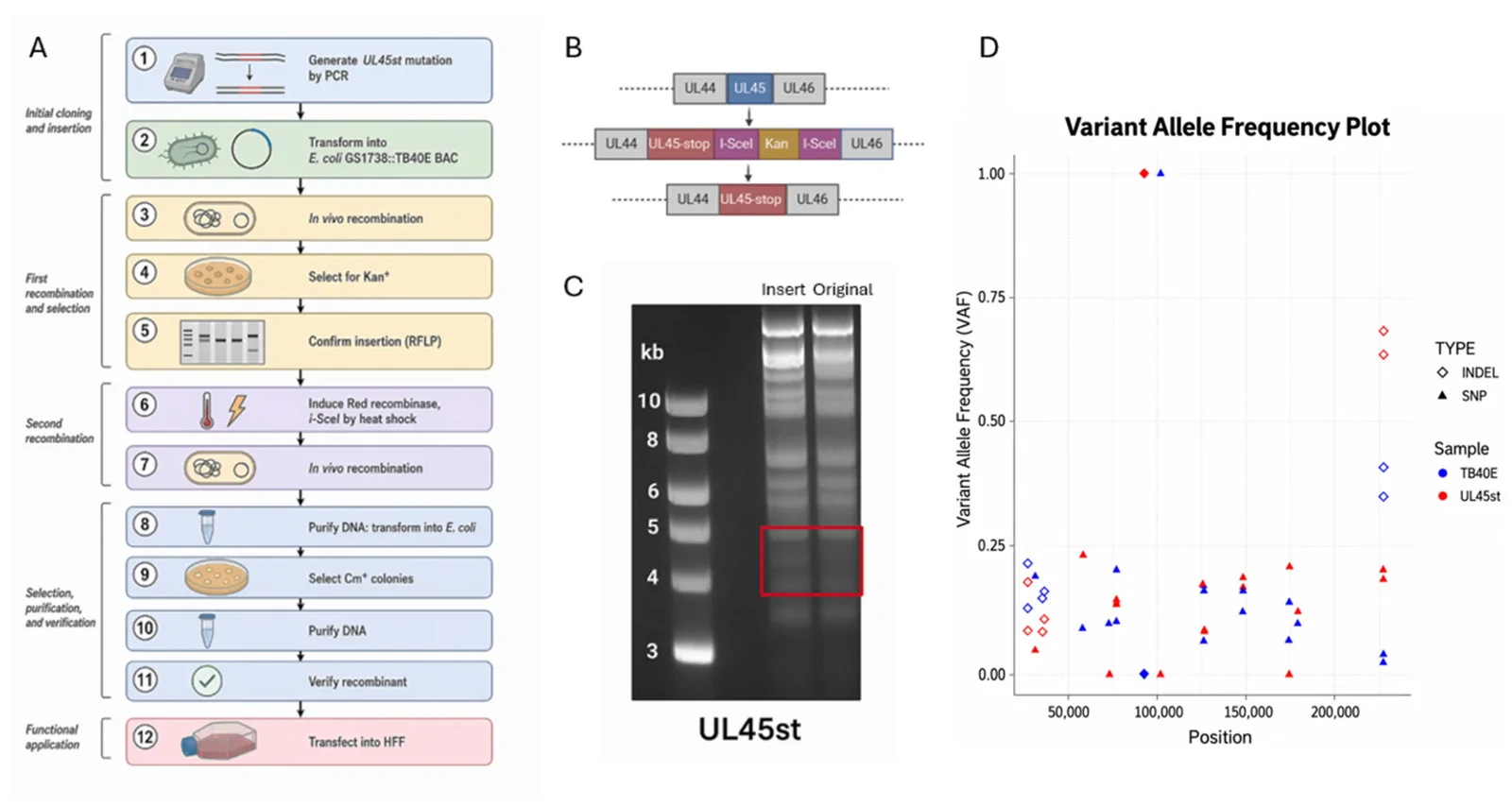
UL45st mutagenesis. A stop codon and a single amino acid deletion were introduced at the start codon of the UL45 gene in TB40E-BAC4, resulting in a stop-frameshift mutation. (**A**) An overview of the two-step Red recombinase mutagenesis method used to create the UL45st mutant. (**B**) A summary of the specific mutation made to generate the UL45st mutant. (**C**) Restriction fragment-length analysis of the intermediate UL45st insert compared to original TB40E BAC DNA. The UL45st insert contained an additional ~4.5 kb DNA fragment containing the kanamycin resistance cassette compared to the original BAC DNA when digested with an *Eco*RI restriction enzyme (indicated by red box). (**D**) After generating infective viral particles from the BAC DNA, viral DNA was extracted from stock virus and sequenced by Illumina next-generation sequencing. Genome reads were then compared to a reference genome (TB40E-BAC4 isolate), and a variant allele frequency (VAF) plot was generated. Wild-type virus and UL45st virus share similar levels of low-frequency mutations (VAF < 0.25). An expected INDEL mutation (VAF = 1) occurs at 93,128 kb in the UL45st mutant with no other mutations in the genome. A previously uncharacterized silent SNP mutation (VAF = 1) was seen in our wild-type virus stock at 101,743 kb (UL48 ORF).

In order to test the role of UL45 in the HCMV response to PCD, mutant virus bearing both a stop codon and a frameshift at the initiation site of the UL45 gene was constructed. To avoid introducing additional exogenous sequences, such as antibiotic resistance genes, this was performed using a two-step Red recombination strategy [35], summarized in a schematic in Figure 1A. Briefly, mutations were introduced into bacterial artificial chromosomes (BACs) containing the TB40E HCMV genome harbored by *Escherichia coli* GS1783 by linear recombination. The first step in generating UL45 mutant BACs was recombination between the parental TB40E-BAC DNA (which confers chloramphenicol resistance) and a linear PCR fragment containing a positive selection marker (*aph*AI, which confers kanamycin resistance), two adjoining I-*Sce*I sites, and a 50 bp duplication around the UL45 start codon. The double-stranded linear fragment was obtained by PCR using the TB40E-BAC DNA as a template and the oligonucleotide primers 5′-AGACACACTTTATTAAAATCTATCTAATTTCTTTGTTCTTTTCCCCGTAAACTTTGACTCCGGCTAACGCGGACGAGGAACAGCGGGtagggataacagggtaatcgattt-3′ and 5′-CGATACACGGGCACCAAGAACACCAGCTGTACCTCGTCCACGTTAGCCGGAGTCAAAGTTTACGGGGAAAAGAACAAAGAAATTAGATAgccagtgttacaaccaattaacc-3′ (integrated DNA Technologies, Coralville, IA, USA) Nucleotides homologous to the TB40E-BAC4 strain are shown in uppercase letters, and those within the *Kan*/I-I*Sce*I cassette are shown in lowercase letters. Linear fragments generated by PCR were transformed into GS1783 containing TB40E-BAC4. Schematics of the initial TB40E, intermediate, and final UL45st BACs are shown in Figure 1B. Recombinants containing the Kan cassette were selected on agar plates containing both chloramphenicol and kanamycin. Antibiotic-resistant clones containing the desired recombinant were confirmed by RFLP analysis. These intermediate BAC constructs were subjected to a second Red recombination event induced by incubating the intermediate BAC construct bacteria at 45 °C for 15 min to induce Red and *Sce*I to delete the kanamycin marker by recombination. The resulting BAC DNA was purified from bacteria and then transformed by electroporation into electrocompetent *E. coli* EL250 cells. Chloramphenicol-resistant transformants were selected and assessed by RFLP analysis (Figure 1C) and Sanger DNA sequencing (Eurofins Genomics, Louisville, KY, USA) to determine deletion of the kanamycin cassette from the intermediate. Recombinant viruses were generated by purifying and transfecting BAC DNA into HFF via PEI transfection. To verify that the UL45st mutant stock did not contain unintended second-site mutations, approximately 1000 base-pair portions surrounding the target mutation of both the wild-type TB40E and UL45st viruses were subjected to Sanger sequencing. Infectious virus titers were determined by plaque assay on HFF. To verify genome integrity, virus stocks were subjected to Illumina next-generation sequencing and variant allele frequency (VAF) analysis (UMMC Molecular and Genomics Core Facility, Jackson, MS, USA).

### 2.4. PBMC Isolation

Discarded blood from deidentified healthy donors was obtained from the UMMC clinical microbiology laboratory. The study was reviewed by the UMMC IRB and found to be “not human research”. All specimens were collected solely for clinical purposes prior to research use; no study procedures were performed on human subjects. PBMCs were separated from the buffy coat within 24 h of obtaining the blood specimens using a density gradient (Ficoll-Hypaque, Sigma-Aldrich) according to the manufacturer’s protocol. Monocytes were then immediately placed in differentiation media in non-tissue-culture-treated plates.

### 2.5. Monocyte Differentiation

Monocytes, obtained as described above, were primed into either M1-like macrophages in the presence of 25 ng/mL GM-CSF or M2-like macrophages using M-CSF (BioLegend) for 6 days. After priming, macrophages were further polarized by incubation with fresh M1 macrophage media containing 25 ng/mL GM-CSF, 10 ng/mL IFN-γ (BioLegend) and 10 ng/mL LPS (Sigma), or M2 macrophage media containing 25 ng/mL M-CSF and 10 ng/mL IL-4 (BioLegend) into M2. Differentiation into either M1 or M2 macrophages was confirmed by differential qRT-PCR analysis with commercially available primer kits for IL1-β, IL-6, IL-10, MRC1, HPRT1, and SDHA (Integrated DNA Technologies) as previously described for determining M1 and M2 macrophage polarization [36].

### 2.6. Macrophage Infection and Relative Numbers of Viable, Metabolically Active Cell Assay

Macrophages were infected with wild-type TB40E virus or UL45st virus at MOI 10. Virus stock was layered on cells with a minimal volume of serum-free medium for 1 h, then aspirated and replaced with complete medium. Relative numbers of viable, metabolically active cells were assessed by measuring intracellular levels of ATP using the Cell Titer-Glo 2.0 luminescent cell viability assay kit (Promega, Madison, WI, USA) according to the manufacturer’s instructions. Luminescence provides a measure of total metabolically active cells present, and was measured on a Bio Tek Cytation 7 microplate reader (Agilent, Santa Clara, CA, USA).

### 2.7. TSV Treatment of Infected Macrophages

Incubation medium was removed at 20 h post infection (hpi) and replaced with complete media containing 30 ng/mL TNF-α (T), 5 µM BV6 (Smac mimetic, S), and 25 µM Z-VAD-FMK (V). Relative numbers of viable, metabolically active cells were assessed as described above.

### 2.8. Transfection of HT29 Cells

HT-29 cells were plated onto 60 mm culture dishes, or 24- or 96-well tray culture dishes. Cells were transfected at 25% confluence with lipofectamine 2000 at a DNA:lipofectamine reagent ratio of 1:10. Transfected cells were harvested at 24 h post transfection for use in either Western blot or necroptosis assays. Cells were transfected at 10%, 25%, 50%, or 75% confluence with DNA to lipofectamine reagent ratios of 1:2, 1:5, or 1:10 when optimizing for transfection efficiency.

### 2.9. HT29 Cell Extrinsic Apoptosis and Necroptosis Assay

HT-29 cells were plated into either 24- or 96-well culture dishes. After transfection or mock transfection for 24 h, media was changed out for complete media containing 30 ng/mL TNF-α (T), 5 μM BV6 (Smac mimetic, S), and/or 25 μM Z-VAD-FMK (V), or combinations of these drugs as described. Cell viability was assessed after 6 h of incubation as described above.

### 2.10. Immunoblots

Samples were solubilized in 4% SDS in the presence of 2-mercaptoethanol and separated on an SDS–polyacrylamide gel, followed by transfer to a polyvinylidene difluoride (PVDF) membrane (BioRad, Hercules, CA, USA). Blots were blocked with 5% non-fat milk in 1% TBST prior to probing with primary antibodies, and subsequent incubation with the HRP-conjugated anti-mouse or anti-rabbit secondary antibody (Sigma), as appropriate. Chemiluminescent signals were detected using SuperSignal West Pico Chemiluminescent Substrate (Thermo Fisher) according to the manufacturer’s instructions. Imaging was performed on a BioRad ChemiDoc MP.

### 2.11. Immunofluorescence Assay

For immunofluorescence analysis (IFA), cells were fixed in 4% paraformaldehyde followed by three washes with phosphate-buffered saline (PBS). The cells were incubated in blocking buffer containing 10% goat serum, diluted in PBS, for 1 h before incubation with primary antibodies. After incubation with mouse anti-IE1/IE2 (Virusys) and rabbit anti-RIPK1 (Cell Signaling), cells were washed three times with PBS, and goat anti-mouse IgG conjugated to Alexa Fluor 568 and goat anti-rabbit IgG conjugated to Alexa Fluor 488 were added. The cells were incubated with 4′,6-diamidine-2′-phenylindole dihydrochloride (DAPI) for 10 min to stain nuclei. Coverslips were retrieved from the wells, mounted on glass slides with a drop of Prolong Gold Antifade mounting medium (Thermofisher), and dried overnight before imaging. Images were captured on a Nikon Eclipse E600 epifluorescence microscope using a Photometics CoolSnap HQ2 CCD camera and processed using MetaView version 7 (Nikon Instruments USA, Melville NY, USA). PowerPoint and Adobe Photoshop Elements 7 were used in the preparation of images, as previously described [27].

### 2.12. Statistical Analyses

All statistical analyses were performed using Prism version 11.1.0 (GraphPad Software Inc., La Jolla, CA, USA). Where data fell into two groups, data were compared for significance using the non-parametric Wilcoxon matched-pairs signed-rank test. Three or more groups of data were compared using ANOVA. All graphs show standard deviation and mean for datasets. *p*-Values (*p*) of <0.05 were considered significant and are indicated with * < 0.05, ** < 0.01, *** < 0.001, **** < 0.0001.

## 3. Results

### 3.1. Generation of a UL45 Stop-Frameshift HCMV (UL45st)

To study the role of UL45 during viral infection, we created a UL45 knockout virus. We introduced a stop-frameshift mutation at the UL45 initiating ATG in TB40E-BAC4 designed to prevent both transcription and translation of the UL45 reading frame (Figure 1) using a two-step markerless Red recombination strategy. Correct insertion of the intended stop-frameshift at the initiation codon in the intermediate BAC construct was confirmed by restriction fragment-length polymorphism (RFLP) analysis: the intermediate construct carried an additional ~4.5 kb band corresponding to the Kan/I-SceI cassette, which was absent in the final UL45st BAC (Figure 1C). To verify genome integrity, virus stocks were subjected to Illumina next-generation sequencing and variant allele frequency (VAF) analysis (Figure 1D). The expected INDEL at position 93,128 kb was present at VAF = 1.0 in UL45st, confirming complete replacement of the wild-type sequence. No other high-frequency mutations were detected in the UL45st genome. A previously uncharacterized silent SNP at position 101,743 kb (within the UL48 ORF) was identified in our wild-type TB40E stock at VAF = 1.0 but was absent from UL45st, indicating it is a stock-specific variant that does not confound comparisons between viruses. Together, these analyses confirm that UL45st carries only the intended mutation and is suitable for phenotypic analysis.

### 3.2. UL45st Exhibits a Replication Defect in Primary Fibroblasts

**Figure 2:**
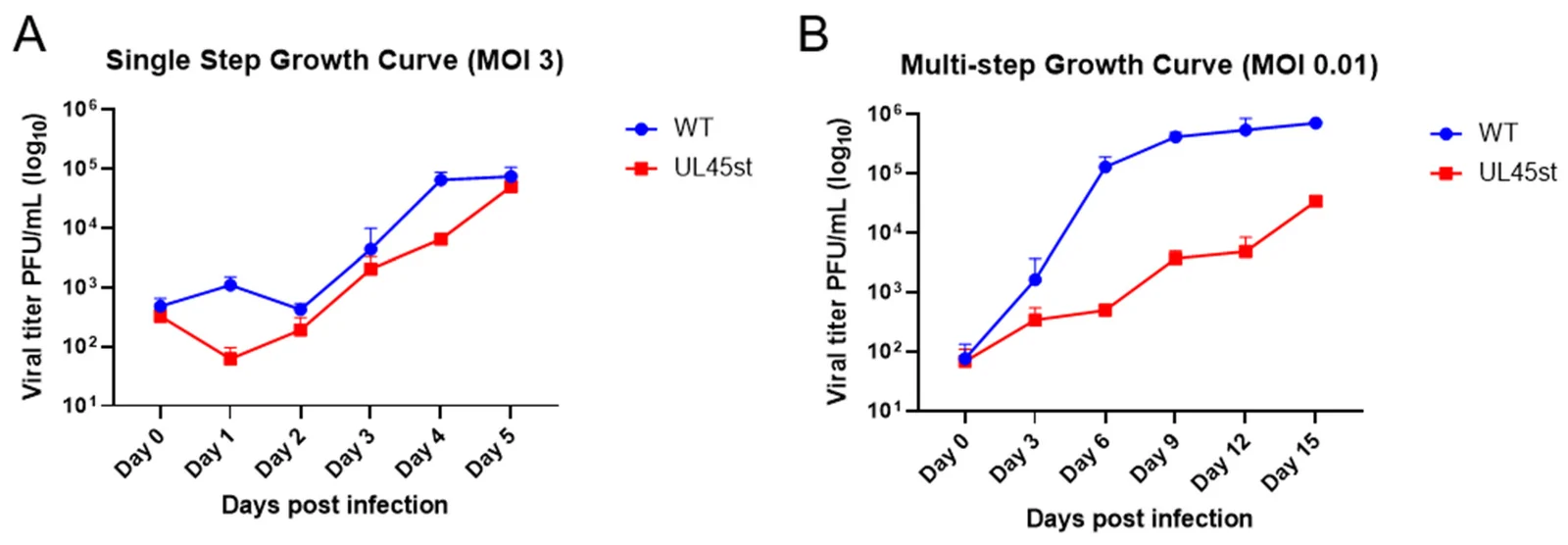
Growth properties of wild-type TB40E virus and UL45st virus in HFF cells at high and low MOI. HFF cells were infected at an MOI of either 3 (panel (**A**)) or 0.01 plaque-forming units (PFUs) per cell (panel (**B**)). Titers of the resulting progeny from both whole-cell lysates and supernatants were determined at the indicated times post infection. The titer at 0 hpi represents the input virus (circles, wild-type TB40E; triangles, UL45st mutant) Each data point is the mean of 3 replicate cultures, with standard deviation bars displayed. Some data sets showed very little variability.

To characterize the growth properties of UL45st, we compared it with the wild-type TB40E strain using single-step (MOI 3, 5 day) and multi-step (MOI 0.1, 15 day) growth curves in primary human foreskin fibroblast (HFF) cells. For single-step analysis, UL45st reached peak titers comparable to wild-type TB40E but with a one-day delay, peaking on day 5 rather than day 4 (Figure 2). Under multi-step conditions, TB40E titers peaked by day 12, consistent with approximately three replication cycles of four days each, while UL45st titers continued to increase through day 15, with a cycle length of approximately five days and a 1–1.5 log_10_ reduction in peak yield. The absence of a proportionate defect under high-MOI single-step conditions suggests the impairment is post entry rather than a defect in virion entry. Assays of viable, metabolically active cells in HFF cultures infected with UL45st showed a modest reduction relative to wild-type HCMV-infected cells. Given the RIPK3 expression in HFFs has been described as negligible [19,36], this effect finding reflects RIPK3-independent death pathways or a minor contaminating RIPK3-expressing cell population rather than necroptosis.

### 3.3. UL45st HCMV Infection Leads to Reduced Viability in Primary Macrophages Compared with Wild-Type Virus

**Figure 3:**
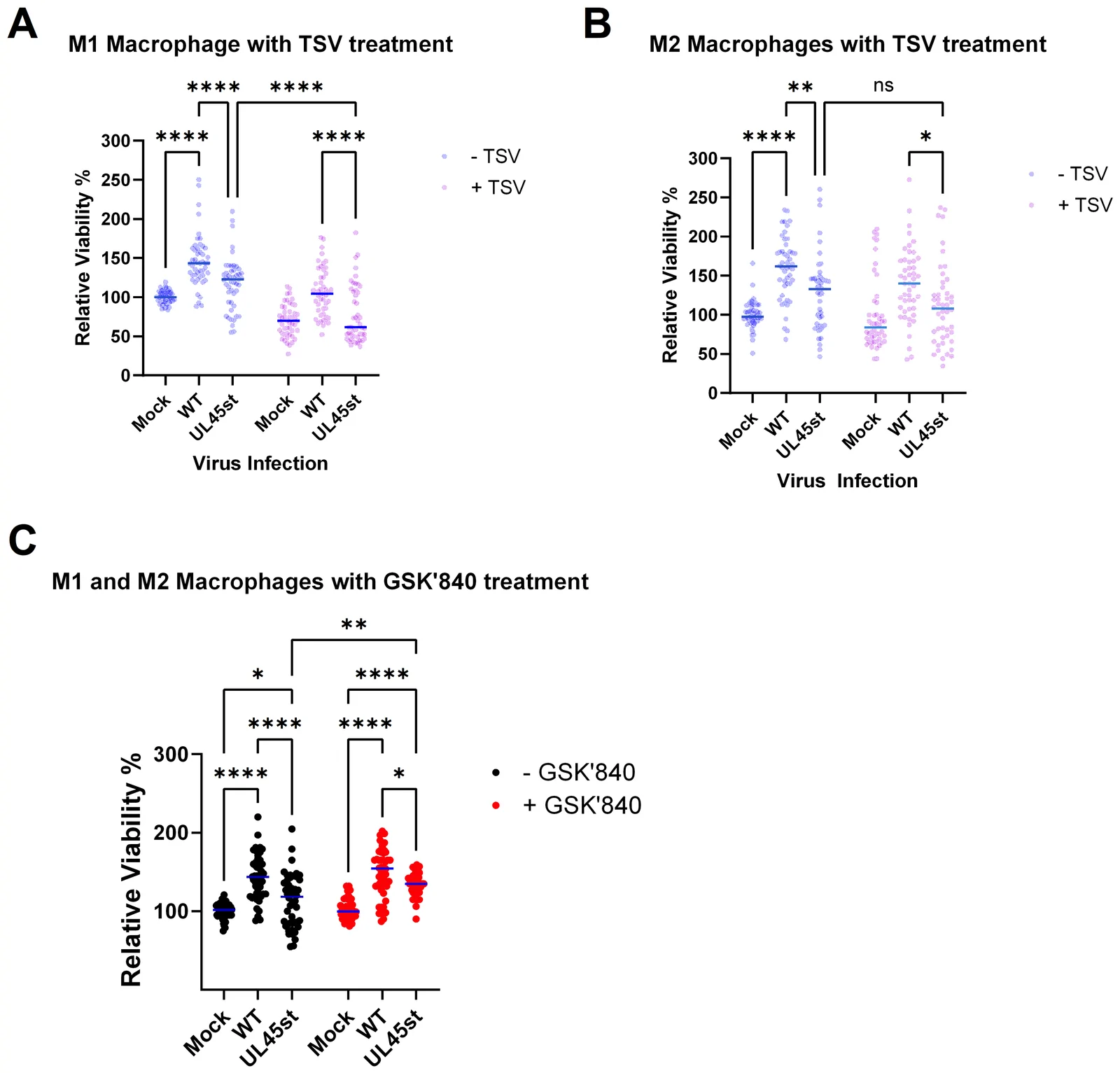
Cell viability of wild-type and UL45st-infected M1 or M2 macrophages. PBMC-differentiated M1 and M2 macrophages from 12 individuals were infected with wild-type TB40E virus and UL45st virus for 24 h. (**A**,**B**) 18 hpi, half the cells were treated with TNF-α, BV6, and Z-VAD-FMK. (**C**) Macrophages from half the donors were treated with GSK840 at the time of infection. Note that cells were differentiated into M1 and M2 macrophages as described previously, but data were pooled for analysis. Cell viability was measured using a Cell Titer Glo ATP assay. All values are normalized to the mock-infected and mock-treated macrophages and show standard deviation and mean for the individuals. For graphs (**A**–**C**), *n* = 12. *p*-Values (*p*) of <0.05 were considered significant and are indicated with ns (not significant), * *p* < 0.05, ** *p* < 0.01, **** *p* < 0.0001.

PBMCs from deidentified individual donors were differentiated into either M1-like or M2-like macrophages. M1-like macrophages were differentiated by sequential GM-CSF priming followed by IFN-γ and LPS stimulation. M2-like macrophages were differentiated by sequential M-CSF priming followed by M-CSF and IL-4 stimulation. M1 and M2 macrophage phenotypes were verified by qRT-PCR analysis of transcripts specific to each cell type. Differentiated macrophages were infected at an MOI of 10 with either wild-type TB40E or UL45st for 24 h, and then viable cell number was assessed by ATP bioluminescence. Consistent with published reports on HCMV-induced pro-survival effect in myeloid cells [24,25], TB40E infection increased macrophage viability by an average of 45% (range 0–60%) compared with uninfected controls from the same donor (Figure 3). By contrast, UL45st-infected macrophages showed an average 30% reduction in viability relative to TB40E-infected cells from the same donor (range 5–50%). This difference was statistically significant across the twelve-donor cohort, although variation between individual donors was observed (Figure S1). Variation may be based on genetic differences between individuals, or may reflect differences in health status, since our samples were discarded after having been drawn for diagnostic purposes at a large academic medical center, and thus were likely to include both well and ill individuals. Infected macrophages from 12 donors were treated with a cocktail containing TNF-α (T), which induces cell death via signaling through the TNFR1 death receptor; BV6 (S), a Smac mimetic and apoptosis-inducing inhibitor of IAP; and Z-VAD-FMK (V), a pan-caspase inhibitor. When this cocktail, termed TSV, was applied at 20 h post infection (hpi), M1-like macrophages from UL45st-infected cultures showed further viability loss compared with TB40E-infected counterparts, while M2-like macrophages were largely insensitive to TSV under these conditions (Figure 3), indicating differential sensitivity to RIPK3-dependent necroptosis between macrophage polarization states. This is also consistent with published differences in necroptotic competence between polarization states. Separately, differentiated macrophages from six donors were treated with GSK840, which has been shown to inhibit necroptosis via the RIPK3/MLKL pathway. GSK840 appears to partially rescue macrophages infected with UL45st, suggesting that GSK840 is complementing a defect in suppression of the RIPK3/MLKL pathway in UL45st-infected macrophages. Note that infections were performed on cells differentiated into either M1 or M2 macrophages, but data were pooled for analysis.

**Figure 4:**
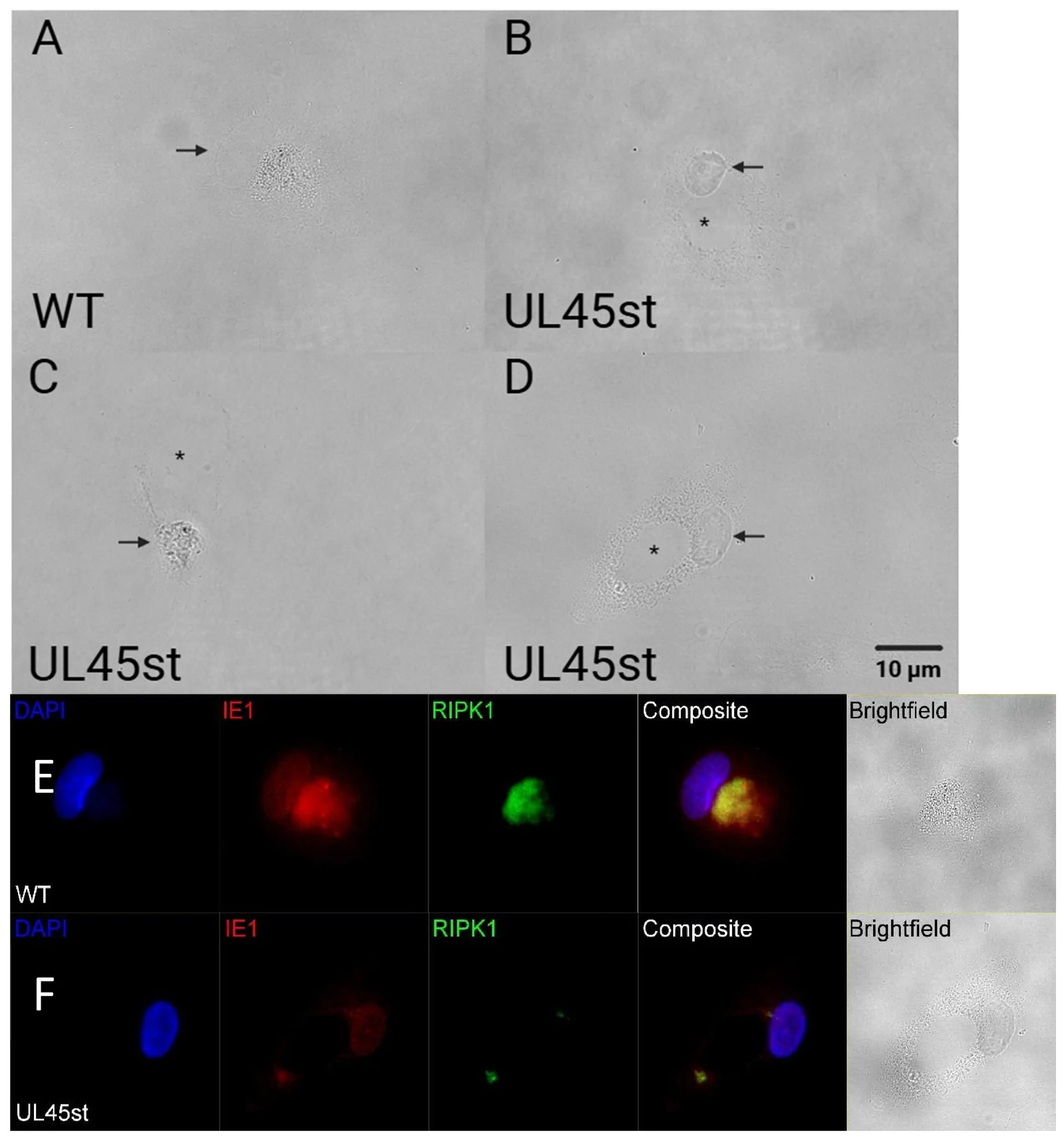
Necrotic-like cell death in UL45st-infected macrophages. Brightfield images of WT and UL45st-infected macrophages at 24 hpi. Nuclei are indicated by arrows. Asterisks (*) indicate large vacuoles in cells (if present). (**A**) Representative TB40E-infected cell. (**B**–**D**) Brightfield images of selected UL45st-infected cells with significant vacuolization. The additional fluorescent panels correspond to panels (**A**,**D**), respectively. (**E**,**F**) Fluorescence imaging of TB40E (**E**) or UL45st (**F**) stained with DAPI (to indicate location of nuclei), and indirect immunofluorescence using antibodies recognizing HCMV IE1 (an infection marker) and host RIPK1 detected using species-specific fluorescently labelled secondary antibodies, along with a brightfield image of the same cell. Note that panels (**E**,**F**) are the same cells as panels (**A**,**D**), respectively, and are shown at the same magnification.

Immunofluorescence assay staining for HCMV IE1/IE2 confirmed productive infection of M1-like macrophages by both viruses. RIPK1 accumulation at the viral assembly complex (VAC) was observed in wild-type-infected macrophages, consistent with prior observations [17]. Strikingly, a substantial fraction of UL45st-infected M1 macrophages at 24 hpi displayed prominent cytoplasmic vacuolization and disrupted cellular membranes not seen in TB40E-infected or uninfected controls (Figure 4). This morphology is consistent with features previously associated with programmed necrosis, which are thought to result from MLKL-mediated plasma membrane disruption, osmotic influx, and organelle swelling [7,9,10]. It should be noted that cytoplasmic vacuolization is not specific to necroptosis, and can accompany other forms of cellular stress, autophagy-related changes, or viral cytopathic effects. Biochemical validation of necroptosis activation in UL45st-infected macrophages, including assessment of RIPK3 and MLKL phosphorylation and MLKL membrane translocation, will be an important objective for future studies.

### 3.4. RIPK3-Dependent Necroptosis Occurs in Primary Macrophages and HT29 Cells, but Not Fibroblasts

**Figure 5:**
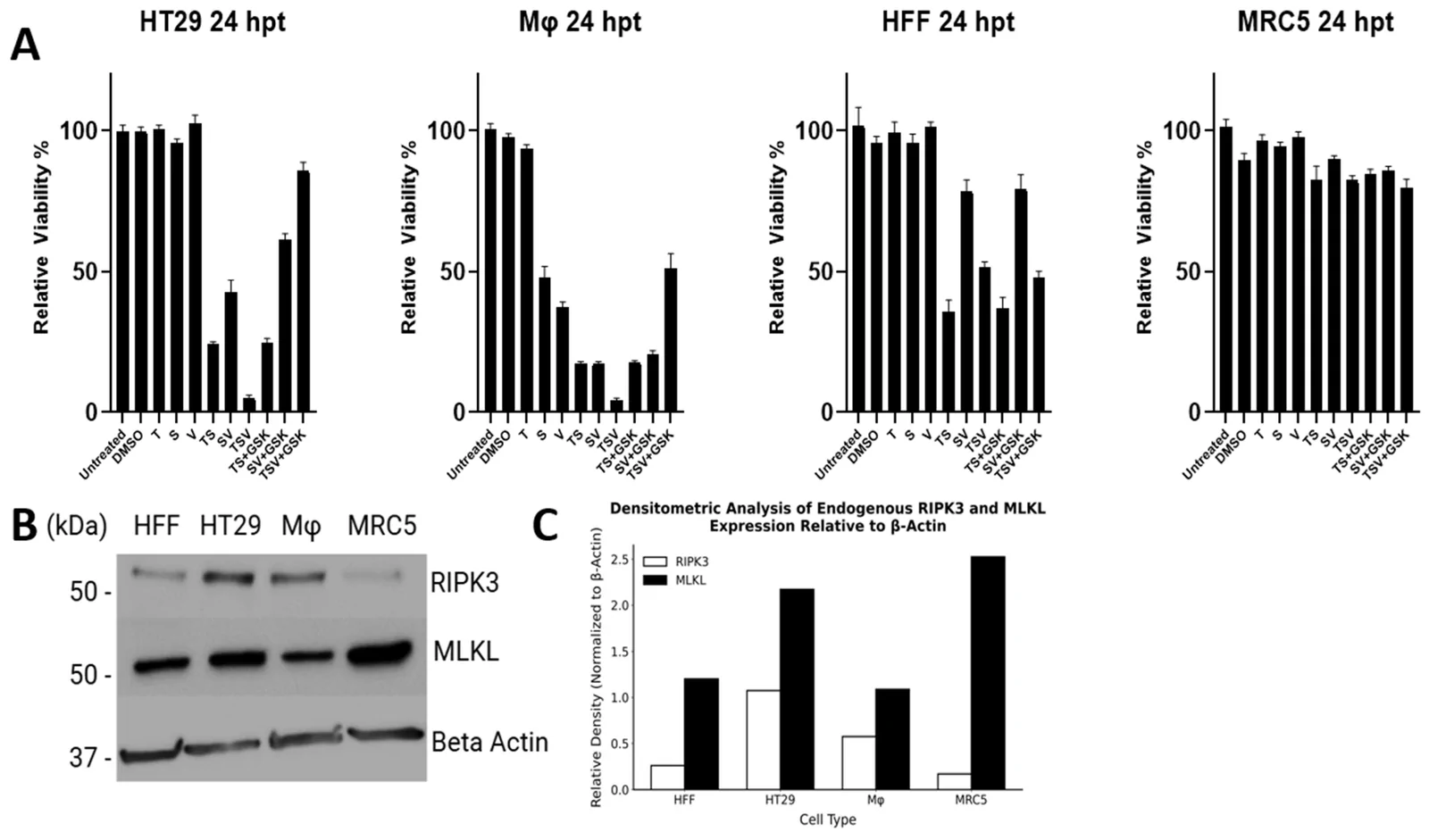
Caspase-dependent and -independent cell death in multiple cell types. (**A**) Human colon carcinoma cell line (HT29), primary PBMC differentiated into M1-like macrophages (Mφ), primary human fibroblasts (HFFs), a and a fetal lung fibroblast cell line (MRC5) were treated for 24 h with a combination of TNF-α: tumor necrosis factor-alpha (T), BV6: a Smac-mimetic (S), Z-VAD-FMK: a pan-caspase inhibitor (V), and/or GSK 840: a RIPK3 inhibitor (GSK). Twenty-four hours post treatment, cell viability was measured by Cell Titer Glo ATP luciferase assay. (**B**) HFF, HT29, M1-like macrophages and MRC5 cells were harvested and tested for endogenous RIPK3 and MLKL levels by Western blot. (**C**) Densitometric analysis of endogenous RIPK3 and MLKL levels to β-actin.

We compared four cell types: primary HFF, human fetal lung fibroblasts (MRC5), PBMC-derived macrophages, and HT29 colorectal adenocarcinoma cells for their sensitivity to chemically induced apoptosis and necroptosis. Cells were treated for 24 h with combinations of TNF-α (T), the Smac mimetic BV6 (S), and the pan-caspase inhibitor Z-VAD-FMK (V), with or without the RIPK3 inhibitor GSK840 (Figure 5A). Relative numbers of viable, metabolically active cells were assessed by ATP bioluminescence. RIPK3 and MLKL expression were confirmed by Western blot (Figure 5B,C). Both fibroblast cell lines were resistant to TSV-induced death, consistent with the low levels of RIPK3 protein detected by Western blot in these cells. Primary M1-like macrophages and HT29 cells, by contrast, expressed robust RIPK3 and MLKL and appeared to undergo substantial cell loss in response to TSV treatment. This loss of viable, metabolically active cells was blocked by GSK840, suggesting a role for RIPK3 in this process. These data suggest that primary macrophages and HT29 cells are preferable to fibroblasts for studying RIPK3-dependent necroptosis and that they were the appropriate systems for the experiments that followed.

### 3.5. UL45 Protein Directly Suppresses Both Apoptotic and Necroptotic Death

**Figure 6:**
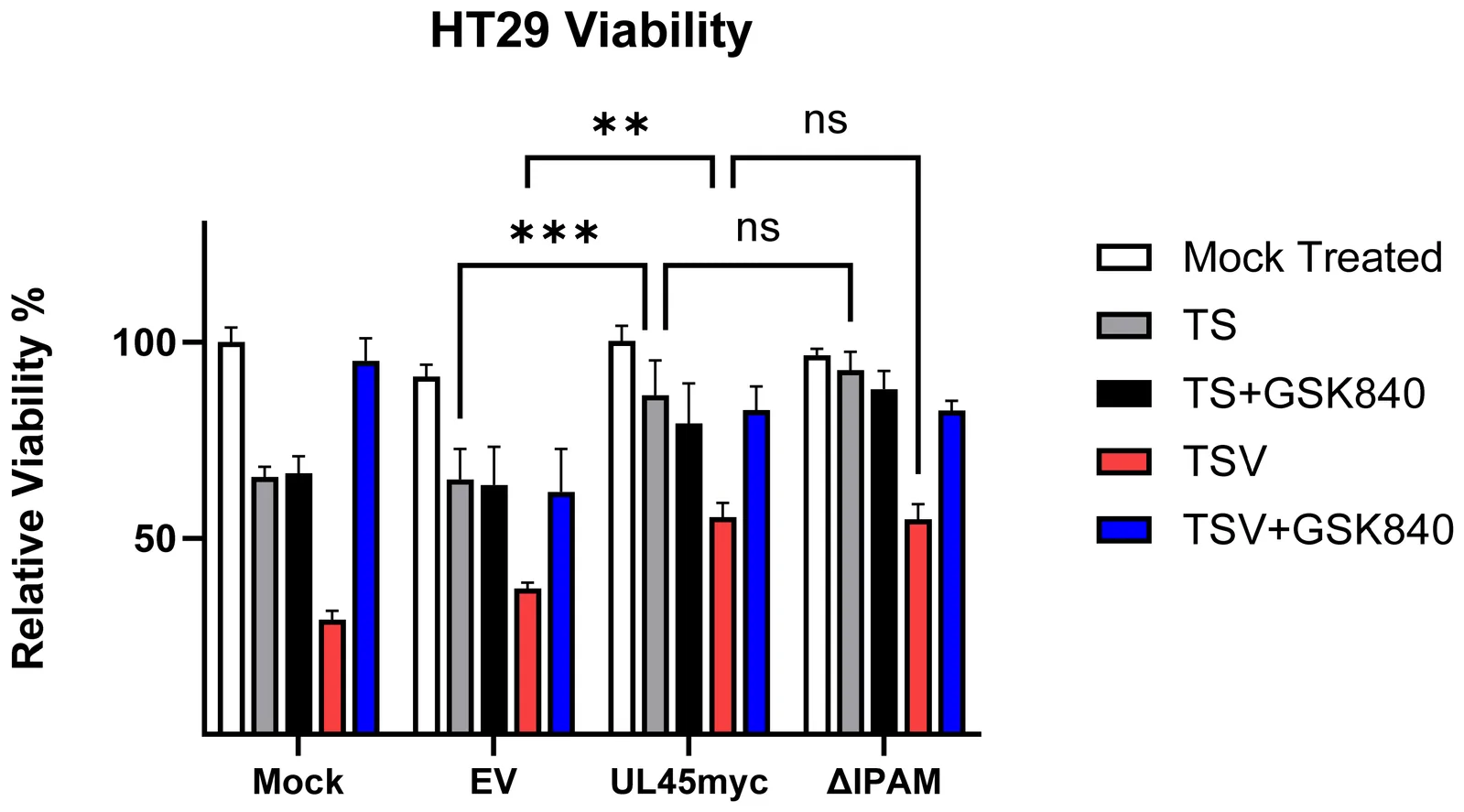
pUL45 partially rescues caspase-independent and caspase-dependent cell death in HT29 cells. HT29 cells were transfected with an empty vector (EV), a plasmid expressing UL45 (UL45myc) or a plasmid expressing UL45 lacking an IPAM domain (ΔIPAM). Twenty-four hours post transfection, media was replaced with cell death media for 24 h that contained the following drugs: TNF-α and BV6 (TS); TNF-α, BV6, and GSK840 (TS+GSK840); TNF-α, BV6, and Z-VAD-FMK (TSV); or TNF-α, BV6, Z-VAD-FMK, and GSK840 (TSV+GSK840). Twenty-four hours post treatment, relative numbers of viable, metabolically active cells were measured with a Cell Titer Glo ATP luciferase assay. *p*-Values (*p*) of <0.05 were considered significant and are indicated with ns (not significant), ** *p* < 0.01, *** *p* < 0.001.

Necroptotic PCD has been extensively characterized in HT-29 cells. To determine whether UL45 has intrinsic PCD-inhibitory activity independent of other viral factors, we transfected HT29 cells with plasmids encoding wild-type UL45 tagged with a Myc epitope (UL45myc) or a deletion mutant lacking the C-terminal IPAM homology domain (UL45ΔIPAM), then subjected cells to chemically induced apoptosis (TS: TNF-α + BV6) or necroptosis (TSV: TNF-α + BV6 + Z-VAD-FMK). The HT29 cell system was selected because these cells are among the few established human cell lines that endogenously express both RIPK3 and MLKL at levels sufficient for canonical necroptosis [15,37,38] (Figure 6). Optimization of transfection conditions increased transfection efficiency to approximately 30%. Expression of UL45myc significantly increased relative numbers of viable, metabolically active cells under both TS and TSV conditions compared with empty vector (expression plasmid only) controls. GSK840 treatment antagonized the effect of TSV; thus, we conclude that TSV-induced death in this system is RIPK3-dependent. UL45ΔIPAM retained protective activity at a level comparable to full-length UL45 under these conditions, indicating that the IPAM domain is not required for protection against PCD by HCMV UL45, in contrast to what was seen in MCMV M45. The protective effect of UL45 against both apoptotic and necroptotic stimuli suggests an upstream mechanism of action common to both apoptosis and necroptosis pathways, rather than targeting pathway-specific effectors.

## 4. Discussion

The central finding of this study is that HCMV UL45 is required for increased macrophage survival during viral infection. This increase in survival was detectable in primary human macrophages, while infections of foreskin and lung fibroblasts produced no comparable phenotype, despite showing a modest growth defect. In addition, the UL45 protein, expressed in the absence of other viral factors, directly suppresses both caspase-dependent apoptotic and RIPK3-dependent necroptotic death in HT-29 cells. Taken together, these observations argue that UL45 functions as an anti-necroptotic factor in macrophages. HCMV research has been performed heavily in fibroblasts and immortalized cell lines, which has likely limited the uncovering of the anti-necroptotic activity of HCMV. Our data show that the cell death pathway antagonized by UL45 depends on RIPK3 since this phenotype was not present with the addition of RIPK3 inhibitor GSK 840 [14]. The same antiviral mechanism that proved essential for MCMV in macrophages in vivo does not function in fibroblast systems where most HCMV anti-PCD research has been conducted. This is not a minor technical caveat; it reflects a fundamental mismatch between the cellular system and the biological question. The appropriate model for studying HCMV necroptosis inhibition is the one in which the relevant pathway is actively engaged—and for a virus that relies on macrophages for dissemination and latency, that model is necessarily the macrophage. The observation that UL45st-infected macrophages show reduced numbers of viable, metabolically active cells by 24 hpi, when viral gene expression is still predominantly in the immediate-early and early phases, is worth examining in the context of UL45’s known classification as a late gene product [22]. One possibility is that UL45 from the incoming virion tegument provides an early burst of anti-necroptotic activity that bridges the gap before late-phase expression begins. This could explain why cells infected with UL45st seem to show the presence of less MIEP signal by immunofluorescence than wild-type TB40E-infected cells. Alternatively, this could simply be due to leakage of soluble cellular contents due to loss of membrane integrity.

HCMV UL45 retains a partially conserved C-terminal IPAM motif [11], which we had theorized to be a necessary domain needed for UL45’s function, since UL45 does not contain the RHIM domain found in MCMV M45. This theory was not supported by our data since the deletion of the IPAM domain did not appear to alter the anti-PCD function of UL45 in the HT29 cell death model. This retention of anti-PCD activity seen in cells expressing UL45 by UL45ΔIPAM in transfected HT29 cells suggests that key cell death signaling proteins like RIPK1, RIPK3, or NEMO may interact with a part of the UL45 molecule outside of the IPAM domain and warrants further study to identify the functional domain(s) of UL45. Conversely, overexpression of UL45myc and UL45ΔIPAM may saturate or bypass IPAM-dependent interactions, possibly masking a contribution of the IPAM domain that may be apparent at physiological expression levels. Further studies examining direct interactions between UL45 and other intracellular PCD mediators are required to confirm the molecular mechanism of UL45 inhibition of necroptosis in vitro. The extremely limited amount of material available from each donor was not sufficient for us to evaluate the mechanism of cell loss in macrophages directly by assessing changes in phosphorylation of intermediates or direct interactions with other components of the PCD pathway and remains a crucial next step in exploration of this topic.

Limitations of the present study include the lack of a commercially available anti-UL45 antibody, which precludes protein-level verification if the UL45 protein is absent from UL45st-infected cells, and limits our ability to verify UL45 interactions using co-immunoprecipitation, since both HT29 and primary macrophages contain Myc, which is the flagging sequence used in our plasmid construct. The differential sensitivity of M1 versus M2 macrophages to TSV-induced necroptosis observed here is consistent with published reports [26,28,39], but warrants more systematic analysis given the known differences in RIPK3 expression and necroptotic pathway activity between these polarization states.

At the broader level, the identification of UL45 as a myeloid-specific anti-necroptotic factor in the human system raises the possibility that pharmacological interference with this function could limit viral persistence. HCMV survival in the myeloid compartment depends on suppressing the very death pathways that innate immune cells use to eliminate infected targets. Compounds that antagonize UL45 function or restore RIPK3-dependent PCD in HCMV-infected macrophages could potentially reduce the viral reservoir that sustains latency and drives reactivation in immunocompromised patients [40].

## 5. Conclusions

HCMV UL45 inhibits RIPK3-dependent cell death in primary human macrophages, a cell type central to viral dissemination and persistence. This function is not apparent in fibroblasts, highlighting the importance of physiologically relevant models for studying viral immune evasion. UL45 expression alone is sufficient to suppress both caspase-dependent and -independent cell death in the well-established HT29 cell death model, which suggests that UL45 is functioning to inhibit an upstream signaling protein common to both apoptotic and necroptotic pathways. Our data uncover a never-before-seen function of UL45 that has the potential to play a key role in establishing and disseminating viral infection in the human host by increasing the lifespan of infected macrophages that may traffic to different niches, carrying virus with them. Further studies are needed to clarify the mechanism of action of UL45 and the specific host cell protein(s) targeted by UL45. UL45 is thus a fruitful target in the quest to prevent novel HCMV infections.

## Data Availability

The data presented in this study are available on request from the corresponding authors.

## Supplementary Materials

The following supporting information can be downloaded at: https://www.mdpi.com/article/10.3390/v18091033/s1, Figure S1: Effect of infection and treatment of macrophages from individual donors. Data were the average and standard deviation of four technical replicates for macrophages from individual male and female donors differentiated into M1 (panel A) or M2 (panel B) macrophages. Overall, the differences in behavior between cells derived from male and female donors was found to be statistically non-significant.

## Abbreviations

The following abbreviations are used in this manuscript:

ATP	Adenosine triphosphate
BAC	Bacterial artificial chromosome
DAPI	4′,6-Diamidino-2-phenylindole
DMEM	Dulbecco’s Modified Eagle Medium
EBV	Epstein–Barr virus
FACS	Fluorescence-activated cell sorting
HCMV	Human cytomegalovirus
HF	Human fibroblast
HFF	Human foreskin fibroblast
HHV	Human herpesvirus
Hpi	Hours post infection
HRP	Horseradish peroxidase
HSV	Herpes simplex virus
IAP	Inhibitor of apoptosis protein
IE1	Immediate-early 1 protein
INDEL	Insertion–deletion
IPAM	Interacting protein aggregation motif
KSHV	Kaposi’s sarcoma-associated herpesvirus
MLKL	Mixed lineage kinase domain-like pseudokinase
MOI	Multiplicity of infection
PBMC	Peripheral blood mononuclear cell
PCD	Programmed cell death
PFU	Plaque-forming unit
PVDF	Polyvinylidene difluoride
RFLP	Restriction fragment length polymorphism
RHIM	Receptor homotypic interaction motif
RIPK1	Receptor-interacting serine/threonine-protein kinase 1
RIPK3	Receptor-interacting serine/threonine-protein kinase 3
RPMI	Roswell Park Memorial Institute medium
RT-PCR	Real-time polymerase chain reaction
SDS	Sodium dodecyl sulfate
SNP	Single-nucleotide polymorphism
TNF-α	Tumor necrosis factor alpha
TSV	TNF-α, Smac mimetic, and Z-VAD-FMK cocktail
UMMC	University of Mississippi Medical Center
VAC	Viral assembly complex
VAF	Variant allele frequency
vICA	Viral inhibitor of caspase-8 activation
vIRA	Viral inhibitor of RIP activation
vMIA	Viral inhibitor of mitochondrial apoptosis
