# Supplementary material for: UL45 of Human Cytomegalovirus Promotes Macrophage Survival and Modulates RIPK3-Associated Cell Death
Source: Viruses. 2026 Sep 17;18(9):1033. doi: 10.3390/v18091033 (PMC13611978; doi:10.3390/v18091033)
Supplement: Supplementary file 1 [file viruses-18-01033-s001.zip › viruses-4554939-supplementary.pdf]

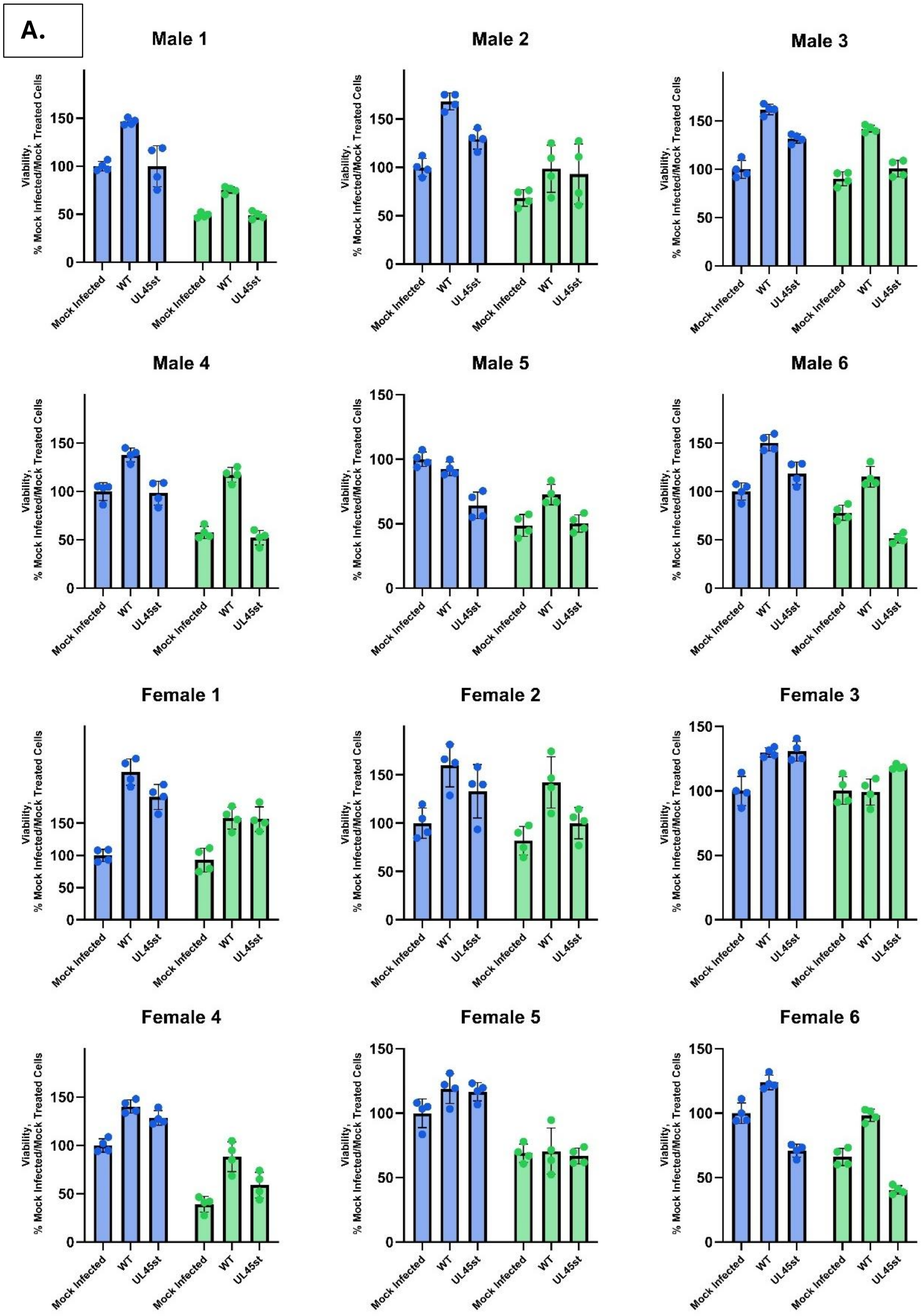
A.
Male 1
Male 2
Male 3
Male 4
Male 5
Male 6
Female 1
Female 2
Female 3
Female 4
Female 5
Female 6
Viability,
% Mock Infected/Mock Treated Cells
Mock Infected
WT
UL45st

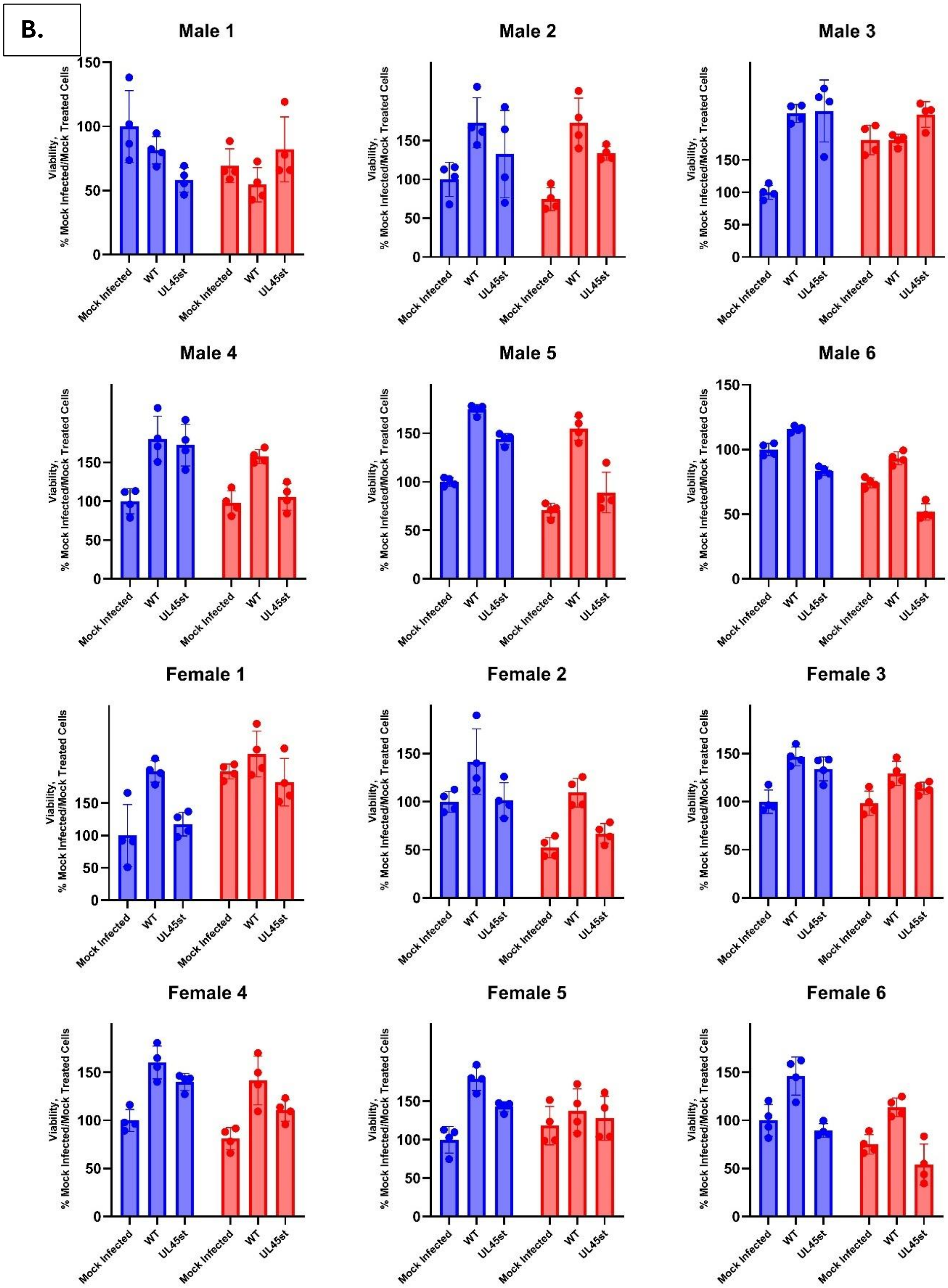
B.
Male 1
Male 2
Male 3
Male 4
Male 5
Male 6
Female 1
Female 2
Female 3
Female 4
Female 5
Female 6
Viability,
% Mock Infected/Mock Treated Cells
0
50
100
150
Mock Infected
WT
UL45st

Figure S1. Effect of infection and treatment of macrophages from individual donors. Data were the average and standard deviation of four technical replicates for macrophages from individual male and female donors differentiated into M1 (panel A) or M2 (panel B) macrophages. Overall, the differences in behavior between cells derived from male and female donors was found to be statistically non-significant.
